# Genome-Wide Identification, Evolution, and Expression Pattern Analysis of the *GATA* Gene Family in Tartary Buckwheat (*Fagopyrum tataricum*)

**DOI:** 10.3390/ijms232012434

**Published:** 2022-10-17

**Authors:** Xin Yao, Meiliang Zhou, Jingjun Ruan, Ailing He, Chao Ma, Weijiao Wu, Dili Lai, Yu Fan, Anjing Gao, Wenfeng Weng, Jianping Cheng

**Affiliations:** 1 College of Agronomy, Guizhou University, Guiyang 550025, China; 2 Institute of Crop Science, Chinese Academy of Agriculture Science, Beijing 100081, China

**Keywords:** *Fagopyrum tataricum*, *GATA*, genome-wide, development and evolution, expression patterns

## Abstract

GATA is a transcription factor that exerts a vital function in plant growth and development, physiological metabolism, and environmental responses. However, the *GATA* gene family has rarely been studied in Tartary buckwheat since the completion of its genome. This study used bioinformatics methods to identify *GATA* genes of Tartary buckwheat and to analyze their subfamily classification, structural composition, and developmental evolution, as well as to discuss the expression patterns of *FtGATA* genes in different subfamilies. The twenty-eight identified *FtGATA* genes in the Tartary buckwheat genome were divided into four subfamilies and distributed on eight chromosomes. One pair of tandem repeat genes and eight pairs of fragments were found in chromosome mapping. Spatiotemporal expression patterns of eight *FtGATA* genes in different subfamilies indicated that the *FtGATA* gene family has regulatory roles in tissue specificity, fruit development, abiotic stress, and hormonal responses. This study creates a theoretical and scientific foundation for further research on the evolutionary relationship and biological function of *FtGATA*.

## 1. Introduction

Transcription factors (TFs) are a class of DNA-binding proteins that regulate gene expression by recognizing specific sequences of target genes and binding to upstream regulatory elements. Therefore, they are also known as trans-acting factors. Transcriptional regulation in eukaryotes is often associated with cis-regulatory elements, such as promoters, enhancers, and silencers [1,2]. TFs are indispensable for plant growth and development and can regulate plant cell differentiation [3], metabolic physiology [4], signal transduction [5], adverse physiology [6,7], and other biological functions. TFs are divided into different family members, including bHLH, MYB, AP2/ERF, NAC, bZIP, and other transcription families according to the level of gene homology, [8]. GATA is a transcription factor with a conserved type IV zinc finger motif. The GATA core base sequence recognizes and specifically binds to the high-affinity (T/A)-GATA-(A/G) sequence, which is composed of one or two highly conserved zinc finger domains (C-X_2_-CX_17–20_-C-X_2_-C; type IVb), and a DNA-binding domain [9]. It is involved in regulating various plant factors in physiological metabolism. The C-X_17–20_ domain of type IV zinc finger structures varies among eukaryotes. Most of the known GATA transcription factors in fungi are C-X_17_, whereas C-X_18_ or C-X_20_ are typically present in plants [10,11].

GATA TFs are ubiquitous in plants and are closely related to plant growth and development, quality, and yield. They regulate the germination and dormancy of plant seeds [12], the formation of organs and morphogenesis [13], the synthesis and accumulation of chlorophyll pigments [14], responses to environmental stress [15], and other biological processes. Phytochrome-interacting factors (PIF) can regulate the expression of GNC (GATA21)- and GNL (GATA22)-related transcription factors that regulate seed germination [16]. Overexpression of *GNC* and *GNL* reduces the rate of seed germination, and their knockdown promotes seed germination in *Arabidopsis*, suggesting that *GNC* and *GNL* play a negative regulatory role in seed germination [17]. *Arabidopsis B-GATA* mutant lines have defective stomatal development in the hypocotyls, whereas stomatal development in the hypocotyls and cotyledons of the overexpressing lines are well-developed [18]. *EurGNC* of *Eucalyptus urophylla* is located in the nucleus and directly binds to the cis-element of the *EurGUN5* promoter, indicating its potential role in the regulation of chlorophyll synthesis [19]. *OsGATA8* overexpression enhances rice tolerance under abiotic stress and improves photosynthesis and yield [20]. *Arabidopsis* GATA TF ZML1 and ZML2 (zinc finger protein expressed in inflorescence meristems such as 1 and 2) mutants were blunted under high irradiance, indicating that GATA TFs are mediated light-responsive pathways [21]. GATA2 is a significant transcriptional regulator that mediates the BR (brassinosteroid)-signaling pathways [22]. BR inhibits *GATA2* transcription through the BR-activated TF BZR1, and light can lead to the accumulation of GATA2 protein and feedback inhibition of GATA2 transcription, suggesting that *GATA2* regulates genes responding to light and BR to affect seedling photomorphogenesis.

Tartary buckwheat (*Fagopyrum tataricum* (L.) Gaertn) is an annual or perennial dicotyledonous crop used for food and forage [23]. It mainly grows in arid and semi-arid high-altitude alpine mountains with low rainfall and a short frost-free period [24,25]. China is ranked among the top producers in the world in terms of planting area and output; current output is second only to that of Russia. Tartary buckwheat is promoted, grown, and used in many countries around the world [26]. It is rich in flavonoid bioactive substances, including natural compounds such as rutin, quercetin, kaempferol, and morin. The rutin content in the roots is significantly lower than that in the shoots [27]. Tartary buckwheat also contains proteins, starch, vitamins, and other nutrients that can relieve cardiovascular sclerosis, diabetes, and other diseases [28]. The fruit is rich in amino acids, and it has a higher total protein content than major grain crops; thus, it can be developed as a healthcare product to relieve various diseases [29,30]. The *GATA* gene family is found in *Arabidopsis* [31], rice [32], *Brachypodium* [33], soybean [34], millet [35], and other crops. However, the Tartary buckwheat *GATA* gene family is not identified, and related studies of this gene family under stress and hormone treatment are rarely reported. This study determines Tartary buckwheat *GATA* family members at the genome level and reports their physicochemical properties, chromosome distribution, cis-acting elements, gene structure, conserved motifs, and phylogenetic trees. Furthermore, tissue-specific and fruit-development expression patterns of the main *FtGATA* family members are analyzed at the grain-filling stage, and differential gene expression analysis is performed under different stresses and hormone treatments to understand their biological function and evolutionary relationship.

## 2. Results

### 2.1. Identification of FtGATA Genes

Twenty-eight *GATA* family members were identified in the Tartary buckwheat genome using two BLAST methods, and they were named *FtGATA01*-*FtGATA28* according to their chromosomal locations (Table S1). Their physicochemical properties were analyzed, including protein length, molecular weight (MW), isoelectric point (pI), subcellular localization prediction, instability index (II), and aliphatic index (AI) (Table S2). FtGATA06 and FtGATA08 were the shortest and longest proteins, with 137 amino acids (15.50 kD) and 524 amino acids (58.15 kD), respectively. The pI ranged from 5.51 (FtGATA19) to 10.55 (FtGATA02), and most had pI < 7, indicating that the gene family was biased towards acidic amino acids. Subcellular localization predicted that 26/28 FtGATAs were located in the nucleus, whereas FtGATA09 and FtGATA13 were supposedly located in the chloroplast. The instability coefficients of 27/28 FtGATA proteins were above 40, indicating that most were unstable, and the least stable was FtGATA08 (73.35). Meanwhile, the FtGATA18 protein was relatively stable, with an instability index below 40. By transmembrane structure prediction, all 28 FtGATA proteins were found to have no transmembrane structure.

### 2.2. Phylogenetic Analysis, Classification, and Multiple Sequence Alignment of Ftgata Proteins

**Figure 1:**
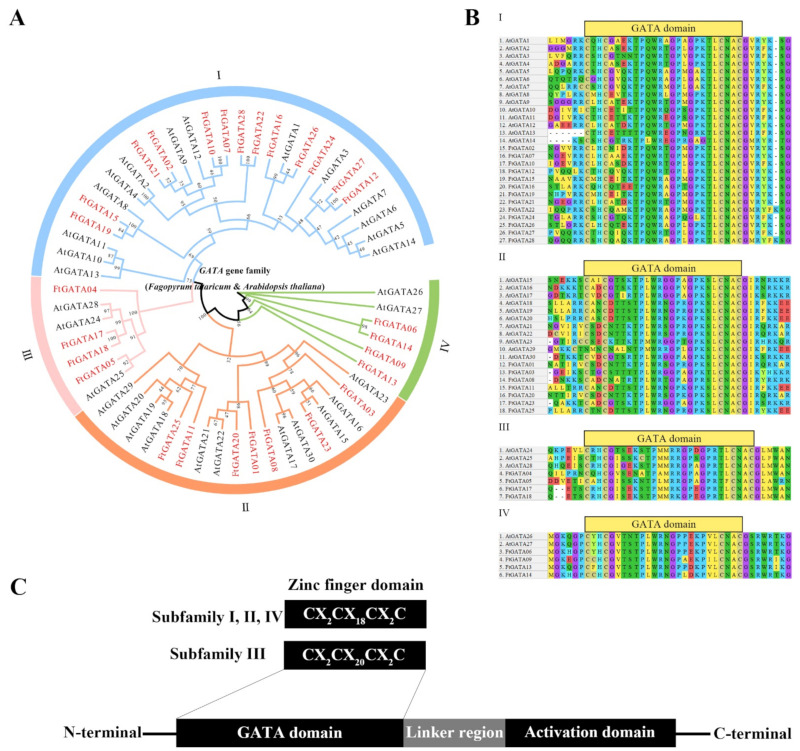
(**A**) Phylogenetic tree presenting the relationship between GATA domains of *Fagopyrum tataricum* and *Arabidopsis thaliana*. (**B**) Multiple sequence alignment of the four phylogenetic GATA domain subfamilies from *F. tataricum* and *A. thaliana*. (**C**) Zinc finger domain and activation domain of the FtGATA factors.

A phylogenetic tree was constructed based on the amino acid sequences of 28 identified FtGATA proteins and 30 AtGATA proteins using TBtools software with a bootstrap value of 1000 (Figure 1A). All 28 identified FtGATA proteins were classified into four subfamilies (I, II, III, and IV) according to the classification of *Arabidopsis* GATAs (Figure 1A). Subfamily I contains the most FtGATA members (13), followed by subfamily II (7), and subfamilies III and IV contain four FtGATAs each (Figure 1A). The evolutionary tree showed that several FtGATAs clustered closely with AtGATA, indicating that these proteins have high homology and similar gene function.

Multiple sequence alignment of the amino acid sequences of FtGATAs and AtGATAs in different subfamilies showed that all FtGATAs contained only one GATA domain (Figure 1B). The GATA domain of subfamily III contains 20 residues in the zinc finger structure (CX_2_CX_20_CX_2_C), and subfamilies I, II, and IV contain 18 residues in the zinc finger structure (CX_2_CX_18_CX_2_C) (Figure 1B,C). The four subfamilies contain highly conserved sequences such as TP, GP, and CNAC; however, there are diversities in GATA domains between different subfamilies and between the same subfamily, such as ET of FtGATA16 in subfamily I, GPR of FtGATA08 in subfamily II, and TF of FtGATA05 in subfamily III (Figure 1B). The zinc finger domain and activation domain of FtGATA factors were connected with the linker region (Figure 1C).

### 2.3. Gene Structures, Conserved Motifs, and Cis-Acting Element Analysis of the Ftgata Gene Family

**Figure 2:**
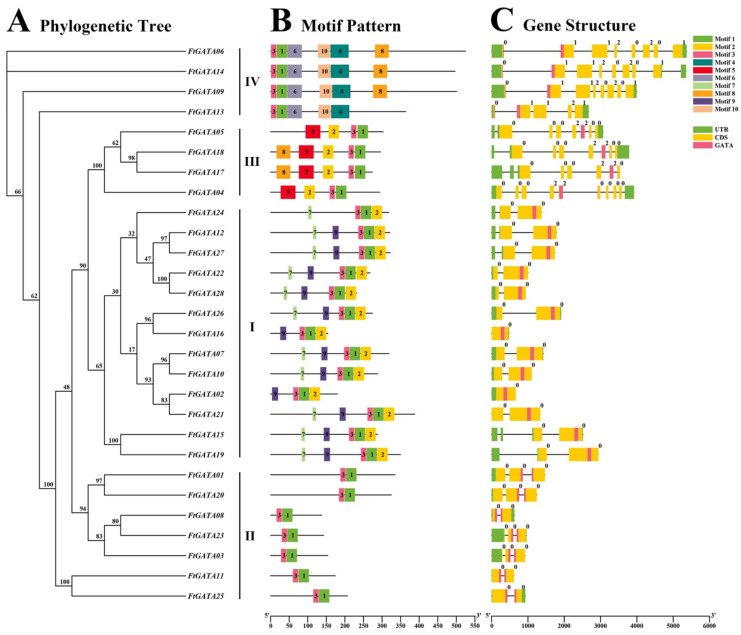
Phylogenetic relationships-, motif distributions-, and gene structure of *GATA* genes in *Fagopyrum tataricum*. (**A**) The phylogenetic tree was constructed with 1000 replicates on each node. (**B**) The 1–10 amino acids motifs in FtGATAs protein are represented with ten different colored boxes, and black lines represent relative protein length. (**C**) The UTR (untranslated region), CDS (coding sequence), GATA domain, and introns are indicated by green squares, yellow squares, pink squares, and black lines, respectively.

TBtools was used to construct 28 FtGATA family evolutionary trees (Figure 2A), and the MEME online program was used to construct 28 FtGATA protein motif structures; motifs were numbered 1–10 (Figure 2B, Table S5). In subfamily I, FtGATA24 did not contain motif 9; FtGATA02 and FtGATA16 did not contain motif 7; and the remaining 10 FtGATA proteins were distributed with motifs 7, 9, 3, 1, and 2. In subfamily II, all seven FtGATAs contain motifs 3 and 1. Motifs 5, 2, 3, and 1 were distributed in most subfamily III members. In subfamily IV, three FtGATAs contain motif 8, whereas FtGATA23 does not. Therefore, there are large differences in motifs between different subfamilies; however, each subfamily contains motifs 3 and 1, and motif 3 is located before motif 1 in the amino acid sequence (Figure 2B). Both subfamilies I and III contain motifs 1, 2, and 3, with motif 2 in subfamily I always located after motif 3 and 1, and motif 2 in subfamily III is located before motif 3 and 1 (Figure 2B).

The gene structure diagram of *FtGATAs* was constructed by comparing the Tartary buckwheat genome data, including the untranslated region (UTR), coding sequence (CDS), GATA domain, and introns (Figure 2C). *FtGATA15* in subfamily I contained the largest number of UTRs (4), whereas the other *FtGATA* genes contained 1 to 3 UTRs. Meanwhile, the *FtGATA04* of subfamily III contains nine CDSs, whereas the *FtGATAs* of subfamily IV contain five to eight CDS segments, and the *FtGATAs* of subfamily I and II each contain one to three CDS. Subfamilies III and IV contained abundant introns: *FtGATA04* contained nine introns, followed by *FtGATA14* (eight), *FtGATA06*, *FtGATA09*, *FtGATA05*, *FtGATA18*, *FtGATA17* (each containing seven introns) (Figure 2C). *FtGATA02* and *FtGATA16* contained few introns in subfamilies I and II, and *FtGATA02* and *FtGATA16* contained no introns, whereas the remainder contained one to three introns and accounted for 67.86% of the 28 *FtGATA* family members.

**Figure 3:**
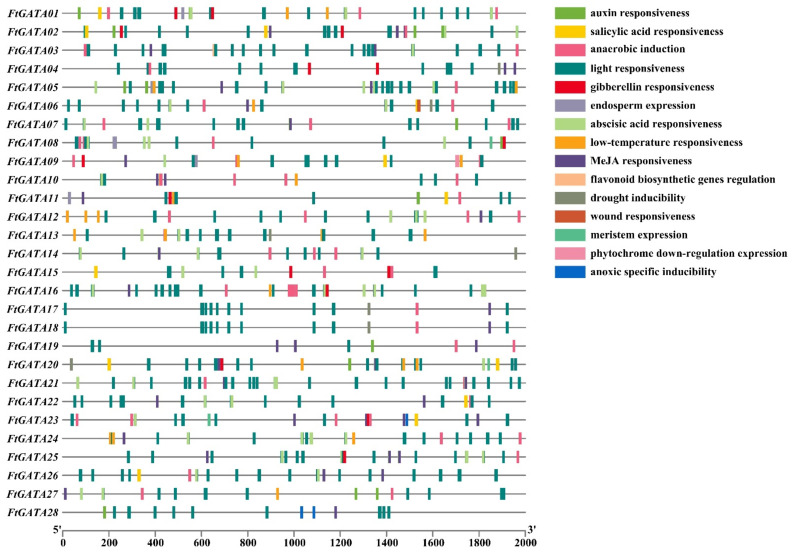
The cis-acting element of the promoter sequence 2000 bp upstream of 28 *GATA* genes in *Fagopyrum tataricum*.

The upstream 2000 bp promoter sequences of the initiation codons (ATG) of 28 *FtGATA* genes were obtained from the genome sequence of Tartary buckwheat, and the cis-acting elements were visualized using TBtools software (Figure 3). The analysis of the promoter sequences of 28 *FtGATA* genes revealed that light- and abscisic acid (ABA)-responsive elements were the most numerous, with 371 and 77 elements, respectively, widely distributed in each promoter sequence. Hormone-related response elements such as abscisic acid (ABA) responsiveness, auxin (IAA) responsiveness, gibberellin (GA) responsiveness, MeJA (methyl jasmonate) responsiveness, and salicylic acid (SA)-responsive elements are distributed in each promoter sequence, with *FtGATA02* and *FtGATA25* promoters containing more hormone-responsive elements (12). This *FtGATA* gene family contains few cis-acting elements related to environmental stress and only includes drunk inducibility (7) and low-temperature responsiveness elements (25). In addition, the promoters of *FtGATA03* and *FtGATA10* contained a flavonoid biosynthetic gene regulation element, indicating that these two genes may directly or indirectly regulate the metabolic function of the flavonoid synthesis pathway (Figure 3).

### 2.4. Chromosomal Distribution-, Gene Duplication-, and Collinear Analysis of Ftgata Genes

**Figure 4:**
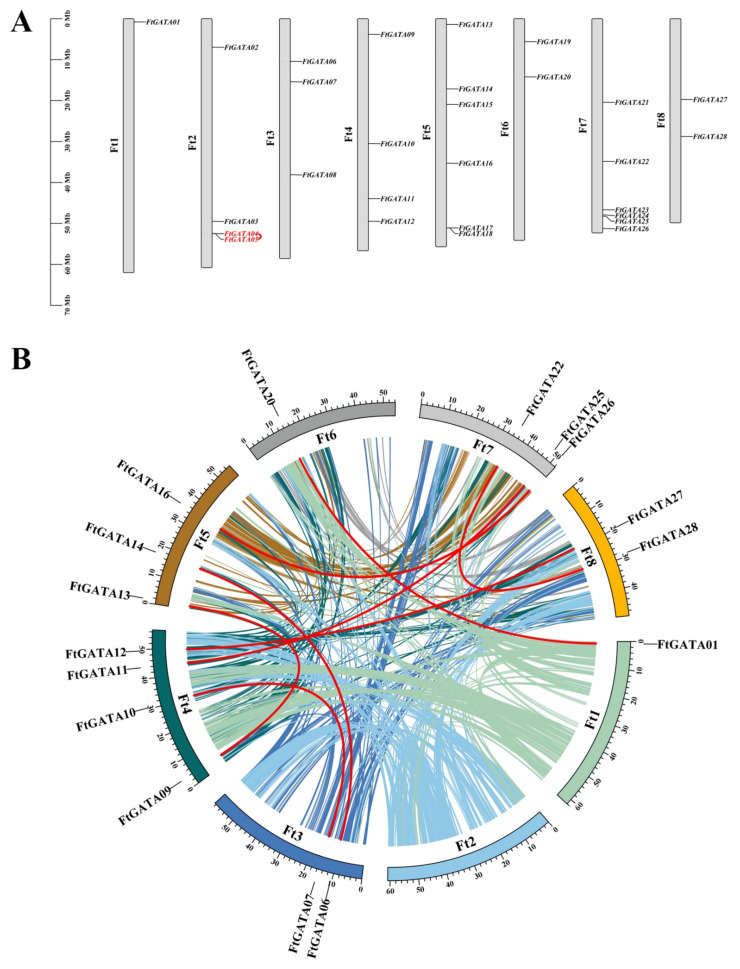
(**A**) Chromosomal distribution of *GATA* genes in *Fagopyrum tataricum*. The gray rectangular bars represent the chromosomes of *F. tataricum*; red fonts and lines represent gene tandem repeats; each corresponding chromosome number is marked on its left side, named Ft1-Ft8, and the 0–70 Mb scale bar on the left-hand side represents chromosome length. (**B**) Chromosome distribution and gene duplication relationship of *GATA* genes in *F. tataricum*. Colored lines indicate the collinear regions in the genomes of *F. tataricum*, and red lines indicate duplicated *GATA* gene pairs. The chromosome number is shown inside each chromosome.

The 28 *FtGATA* genes were mapped to the corresponding Ft1–Ft8 chromosomes (Figure 4A). There were different numbers of *FtGATA* genes on each chromosome: *Ft1* contained only one gene (*FtGATA01*, 3.57%), followed by *Ft6* (*FtGATA19* and *FtGATA20*, 7.14%), *Ft8* (*FtGATA27* and *FtGATA28*, 7.14%), then *Ft5* and *Ft7*, which contained four genes (approximately 14.29%). Repeats in which one or more nucleotides in DNA are connected before and after are defined as tandem repeats, or they can also be called tandem repeats when two or more genes overlap within a 200 kb chromosome [36]. Only one pair of genes (*FtGATA04* and *FtGATA05*) in the *FtGATA* gene family had tandem duplications on the *Ft2* chromosome (Figure 4A, Table S6), and both of these genes belong to subfamily III.

Fragment repeat analysis on 28 *FtGATA* genes by BLASTP and MCScanX identified 16 similar loci and eight pairs of fragment duplication events: *FtGATA06/FtGATA14*, *FtGATA07/FtGATA10*, *FtGATA09/FtGATA13*, *FtGATA11/FtGATA25*, *FtGATA12/FtGATA27*, *FtGATA16/FtGATA26*, *FtGATA20/FtGATA01*, and *FtGATA22/FtGATA28* (Figure 4B, Table S7). Four pairs of *FtGATA* genes belonged to subfamily I, and subfamilies II and IV each contained two pairs of GATA duplication events. The *FtGATA* gene family was unevenly distributed in the eight linked regions (LG) of Tartary buckwheat, and LG4 contained the most GATA genes (4/16, 25%), followed by LG5 (3/16, 18.75%), and LG7 (3, 18.75%).

**Figure 5:**
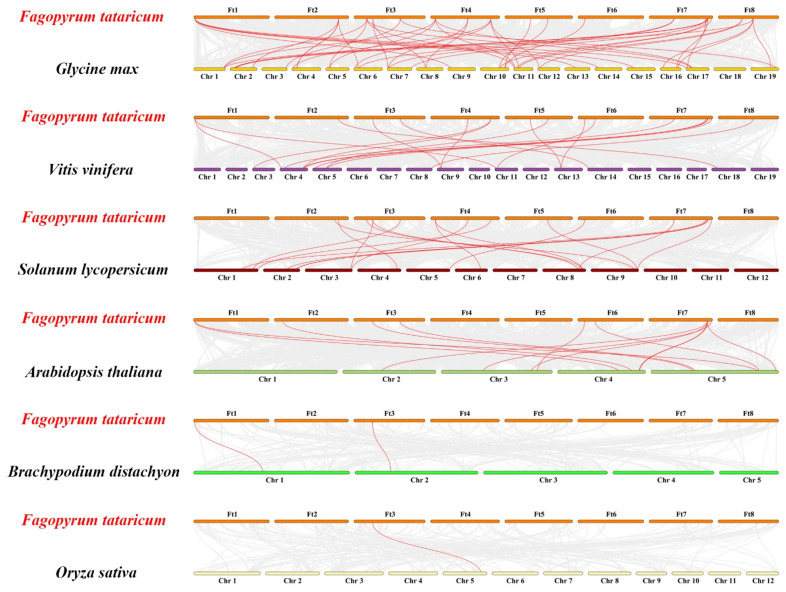
Collinear analysis of GATA genes between *Fagopyrum tataricum* and six different plants (*Glycine max*, *Vitis vinifera*, Solanum lycopersicum, Arabidopsis thaliana, *Brachypodium distachyon*, and *Oryza sativa*). The gray lines between *F. tataricum* and other plants represent collinearity in wide regions of the genomes, and red lines show the orthologous relationship of GATA genes. Ft represents the chromosome number of *F. tataricum*, and Chr represents the chromosome number of the six other plants.

A collinear map of Tartary buckwheat and six different plants, including two monocots (*Brachypodium distachyon* and *Oryza sativa*) and four dicots (*Glycine max*, *Vitis vinifera*, *Solanum lycopersicum*, and *Arabidopsis thaliana*) was constructed (Figure 5). The collinear relationship between the *FtGATA* gene family and dicotyledonous plants was stronger than that of monocotyledonous plants. The most collinear gene pairs were between *Glycine max* and *FtGATA* (46 pairs), followed by *Vitis vinifera* (18 pairs) and *Solanum lycopersicum* (18 pairs). However, the *FtGATA* gene has weak collinear relationships with monocotyledonous plants; there were only two pairs of collinearity with *Brachypodium distachyon*, followed by *Oryza sativa* (one pair). *FtGATA07* was collinear with both the dicotyledonous and monocotyledonous plants (Figure 5 and Table S8). *FtGATA08*, *FtGATA24*, *FtGATA25*, and *FtGATA26* had collinear gene pairs in dicotyledonous plants. This suggests that *FtGATA07*, *FtGATA08*, *FtGATA24*, *FtGATA25*, and *FtGATA26* had homologous genes before dicotyledonous plant differentiation. However, *FtGATA11*, *FtGATA13*, *FtGATA14*, *FtGATA17*, *FtGATA18*, and *FtGATA22* did not have collinear homologous genes in the six plants; this suggested that these genes may have formed after the plant had differentiated.

### 2.5. Evolutionary Analysis of FtGATA Proteins and GATA Proteins of Different Plants

**Figure 6:**
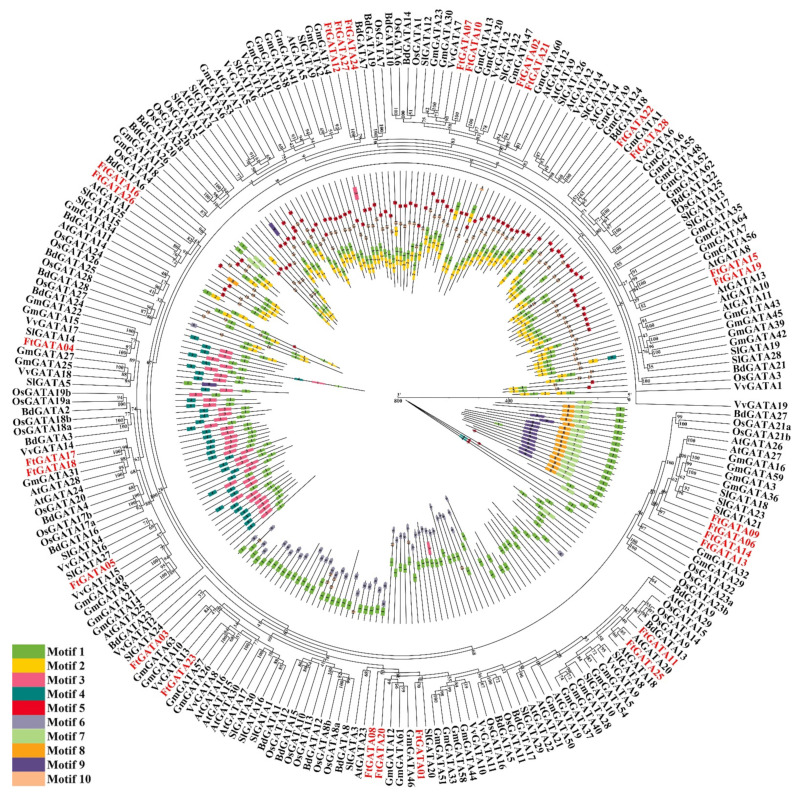
Phylogenetic relationship and conserved motifs of GATA protein in *Fagopyrum tataricum* and six plants (*Glycine max*, *Vitis vinifera*, *Solanum lycopersicum*, *Arabidopsis thaliana*, *Brachypodium distachyon*, and *Oryza sativa*). The red fonts represent the 28 *FtGATA* genes identified in this paper. The colored legends represent 10 motifs, the outer part of the circle represents the phylogenetic tree of GATA proteins, and the inner part of the circle represents different conserved motifs and protein lengths.

A developmental phylogenetic tree with 1–10 conserved motifs was constructed using the identified FtGATA amino acid sequences and six plant GATA amino acid sequences to further explore the evolutionary relationship between FtGATA proteins (Figure 6, Table S9). The distribution of FtGATA proteins in the phylogenetic tree species was relatively scattered, and most of the FtGATA proteins clustered closely with *Glycine max* and *Solanum lycopersicum*. This indicated that FtGATAs have close genetic relationships with GATAs from dicotyledonous plants. All GATA protein sequences contained motif 1, and each subfamily contained similar motif components for different groups (Figure 6). For example, subfamily I contains motif 10–1–2, and subfamily III contains motifs 4–3–1, which indicates that these proteins have similar structures and functions. There were also different proteins with different motifs in the same group. For example, FtGATA25 of subfamily II only has motif 1, whereas FtGATA14 of subfamily IV has motif 5.

### 2.6. Expression Patterns of FtGATAs in Different Plant Tissues and Fruit Development

**Figure 7:**
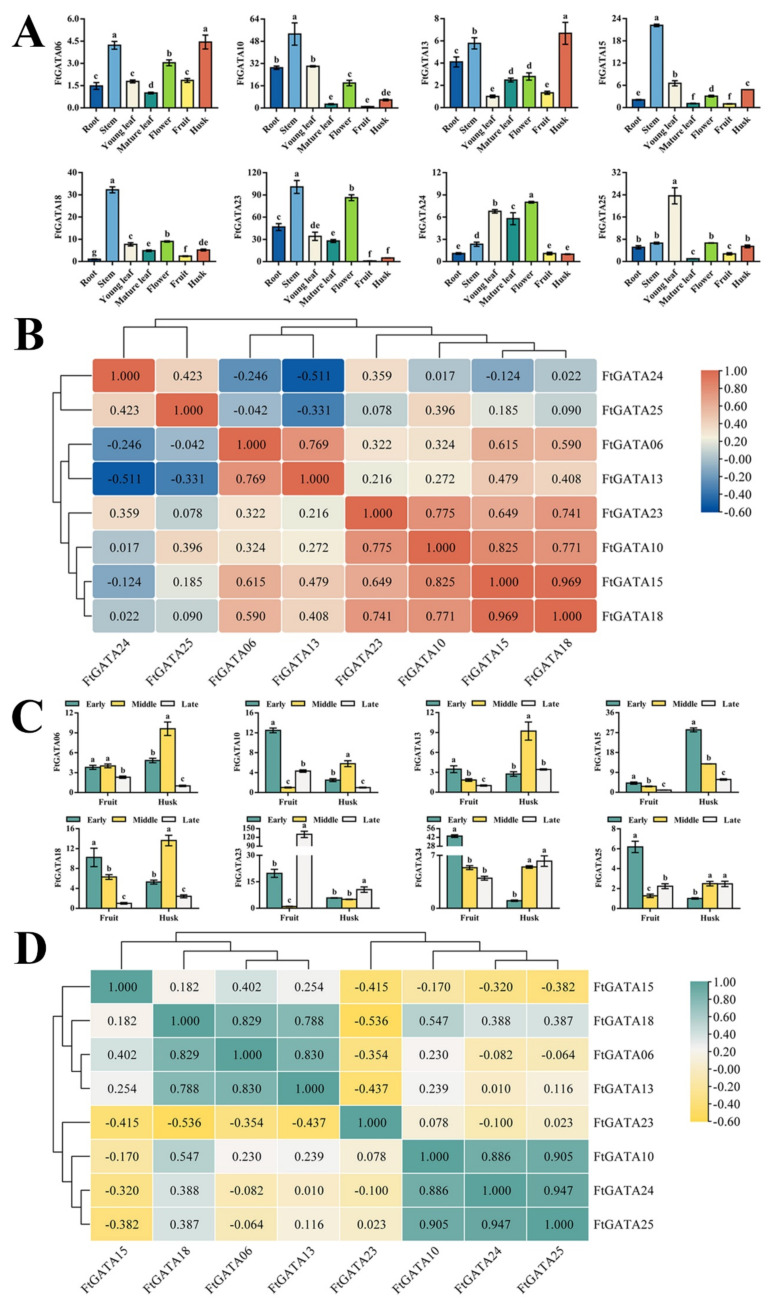
Relative expression levels and correlation hierarchical cluster analysis of 8 *Fagopyrum tataricum GATA* genes (*FtGATA06*, *FtGATA10*, *FtGATA13*, *FtGATA15*, *FtGATA18*, *FtGATA23*, *FtGATA24*, and *FtGATA25*) in tissues and during fruit development. (**A**) Expression patterns of eight FtGATA genes at the mid-grain filling stage in roots, stems, young leaves, mature leaves, flowers, fruit, and husks. (**B**) Correlation analysis between their expression in different tissues at the mid-grain filling stage. (**C**) Expression patterns of eight *FtGATA* genes in the fruit and husk during early, middle, and late grain-filling stages. (**D**) Correlation analysis between their expression in the fruit and husk during the grain-filling stage. Column chart values are expressed as the mean ± SD according to Duncan’s multiple comparisons; different lowercase letters represent significant differences among different treatments (*p* < 0.05). Positive and negative numbers in the schematic graph of correlation represent positive and negative correlation, respectively; larger values indicate stronger correlations.

Eight FtGATA genes from different subfamilies were selected to analyze the relative expression levels of six tissues (root, stem, young leaf, mature leaf, flower, fruit, and husk) in the middle stage of grain filling (Figure 7A). The relative expression levels of most *FtGATA* genes (*FtGATA06*, *FtGATA10*, *FtGATA13*, *FtGATA15*, *FtGATA18*, and *FtGATA23*) was high in the stems, and some genes were highly expressed in flowers (*FtGATA23* and *FtGATA24*) and husks (*FtGATA06* and *FtGATA13*), but the overall expression levels of *FtGATA* genes in fruit and mature leaves was low. Correlation analysis of *FtGATA* genes from seven tissues at the grain-filling stage showed that *FtGATA10*, *FtGATA18*, and *FtGATA15* were of a significantly positive correlation (*p* < 0.05), *FtGATA13* and *FtGATA24* were of a significantly negative correlation (*p* < 0.05), and *FtGATA10*, *FtGATA18*, and *FtGATA24* showed low correlations (Figure 7B).

Tartary buckwheat seeds are rich in many nutrients, particularly bioactive flavonoids. Therefore, the relative *FtGATA* expression levels in its fruit and husk during early, middle, and late grain filling were analyzed (Figure 7C). The relative expression level of most *FtGATA* genes was higher in the early stage of grain filling (*FtGATA10*, *FtGATA13*, *FtGATA15*, *FtGATA18*, *FtGATA24*, and *FtGATA25*), among which *FtGATA24* expression was the highest, followed by relatively high expression in the husk in the middle stage of grain filling (*FtGATA06*, *FtGATA10*, *FtGATA13*, and *FtGATA18*), whereas the overall expression level in the husk during the early stages of grain filling was low. *FtGATA24*, *FtGATA25*, and *FtGATA10* were of a significantly positive correlation (*p* < 0.05), *FtGATA13* and *FtGATA18* were of a significantly negative correlation (*p* < 0.05), and *FtGATA13* and *FtGATA24* were weakly correlated (Figure 7D).

### 2.7. Expression Patterns of FtGATA Genes in Response to Abiotic Treatments

**Figure 8:**
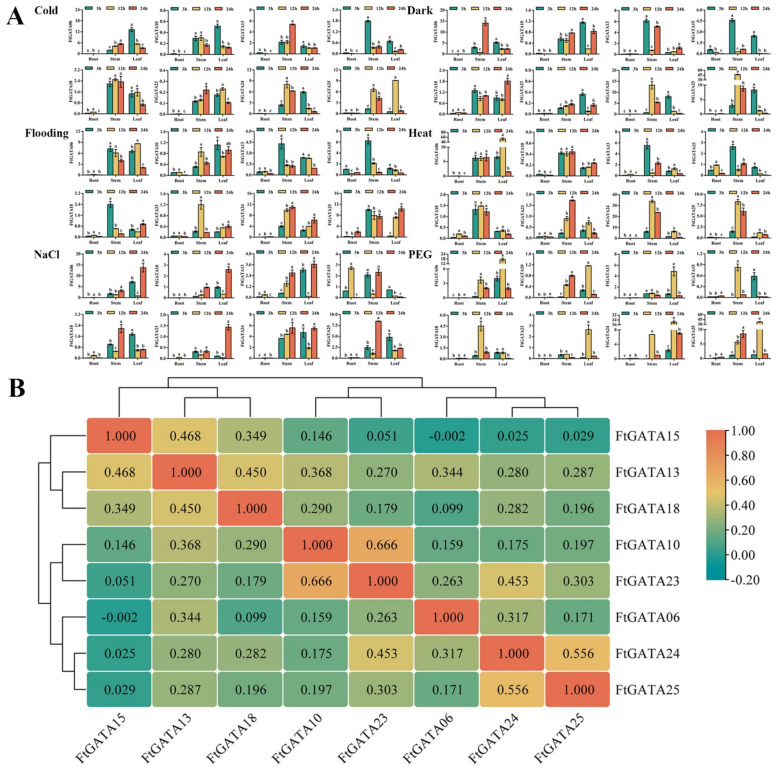
Spatiotemporal relative expression levels of eight *Fagopyrum tataricum GATA* genes (*FtGATA06*, *FtGATA10*, *FtGATA13*, *FtGATA15*, *FtGATA18*, *FtGATA23*, *FtGATA24*, and *FtGATA25*) under different stresses (cold, dark, flooding, heat, NaCl, and PEG) at the seedling stage and correlation hierarchical cluster analysis between their expression. (**A**) Expression patterns of eight *GATA* genes at 3 h, 12 h, and 24 h in roots, stems, and leaves. (**B**) Correlation analysis between their expression. Column chart values were expressed as the mean ± SD according to Duncan’s multiple comparisons; different lowercase letters represent significant differences among different treatments (*p* < 0.05). Positive and negative numbers in the schematic graph of correlation represent positive and negative correlation, respectively; larger values indicate stronger correlations.

Relative expression analysis of *FtGATA* under six different abiotic stress treatments was performed to preliminarily explore the regulatory mechanism of *FtGATA* under abiotic stress (Figure 8A). The *FtGATA15* expression level was higher in stems and leaves after 3 h of treatment, and the expression levels of *FtGATA23* and *FtGATA25* genes of subfamily II significantly increased in leaves under cold and PEG (polyethylene glycol) stress for 12 h. The overall trends of *FtGATA06* and *FtGATA13* under different stress treatments were similar. For example, the leaf expression was the highest at 12 h following PEG treatment, and at 3 h and 24 h after NaCl treatment. *FtGATA18* did not significantly change in stems under cold and heat treatments, suggesting that it might not have a relevant regulatory effect on stems under cold and heat treatments. Correlation analysis of the related expression under six abiotic stress conditions indicated that *FtGATA10*, *FtGATA13*, *FtGATA18*, *FtGATA23*, *FtGATA24*, and *FtGATA25* were positively correlated (*p* < 0.05), whereas *FtGATA06* and *FtGATA15* were insignificantly negatively correlated (Figure 8B).

### 2.8. Expression Patterns of FtGATA Genes in Response to Hormone Treatments

**Figure 9:**
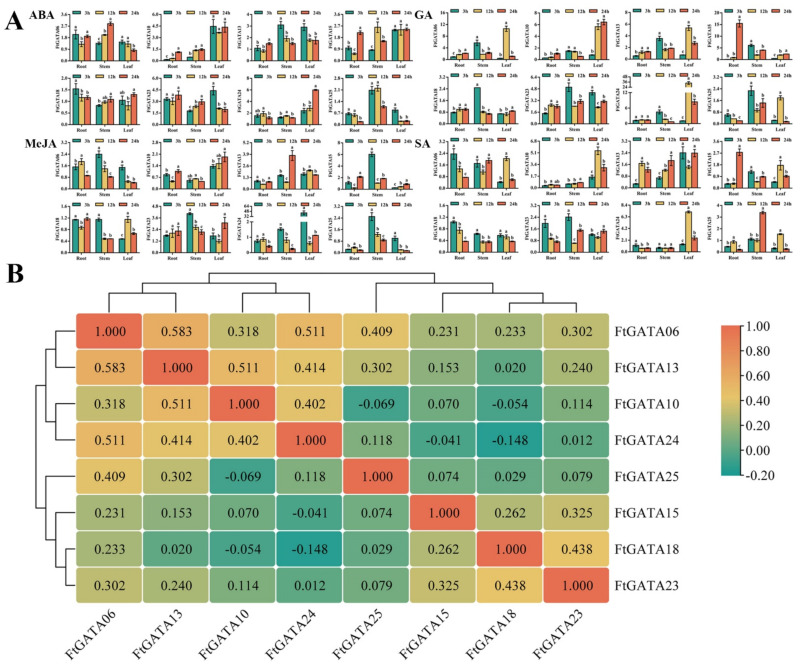
Spatiotemporal relative expression levels of eight *Fagopyrum tataricum GATA* genes (*FtGATA06*, *FtGATA10*, *FtGATA13*, *FtGATA15*, *FtGATA18*, *FtGATA23*, *FtGATA24*, and *FtGATA25*) under different hormone treatment (abscisic acid, ABA; gibberellin, GA; methyl jasmonate, MeJA; and salicylic acid, SA) at the seedling stage and correlation hierarchical cluster analysis between their expression. (**A**) Expression patterns of eight *GATA* genes at 3 h, 12 h, and 24 h in roots, stems, and leaves. (**B**) Correlation analysis between their expression. Column chart values were expressed as the mean ± SD according to Duncan’s multiple comparisons; different lowercase letters represent significant differences among different treatments (*p* < 0.05). Positive and negative numbers in the schematic graph of correlation represent positive and negative correlation, respectively; larger values indicate stronger correlations.

A previous analysis of cis-acting elements of the *FtGATA* gene family showed that hormone response elements are distributed in each promoter sequence (Figure 3). Therefore, this study analyzed the relative expression levels of *FtGATA* genes in different subfamilies under different hormone treatments (Figure 9A,B). The expression levels of genes other than *FtGATA10* were higher in the stem under 3 h GA treatment, and the expression levels of genes other than *FtGATA10* and *FtGATA13* were higher in the stem under 3 h MeJA treatment. This indicated that some genes were upregulated in the stem. The expression levels of *FtGATA10* and *FtGATA15* in subfamily I were not significantly different in leaves under ABA treatment. *FtGATA18* expression from subfamily III was the highest in roots treated with ABA, MeJA, and SA for 3 h. Correlation analysis of expression data from the eight genes under different hormone treatments showed that *FtGATA06*, *FtGATA10*, *FtGATA13*, and *FtGATA24* were all significantly positively correlated, whereas *FtGATA24* was negatively correlated with *FtGATA18*, *FtGATA24*, and *FtGATA25*, but insignificantly correlated between *FtGATA23* and *FtGATA24* (Figure 9B).

## 3. Discussion

### 3.1. Characteristics of FtGATA Genes

The physicochemical properties, protein structure, evolutionary relationship, and spatiotemporal expression of the *FtGATA* gene family were systematically analyzed, and 28 *FtGATA* genes were identified. FtGATAs ranged from 137 to 524 amino acids (15.50–58.15 kD) (Table S2), indicating that they have large structural differences that may be related to genome size and species evolution. The pI of 16/28 (57.14%) *FtGATA* genes was under seven (Table S2), indicating that most *FtGATA* genes were rich in acidic amino acids. However, this was contrary to the results of some GATA transcription factors [35,37,38], and the findings for different protein families in Tartary buckwheat were consistent [39]. This indicated that the pI of the same gene family is different for different species, and the pI of the same species may be the same for different gene families. Tartary buckwheat is a plant with more alkaline substances that can be processed and made into products such as Tartary buckwheat noodles and tea. However, the *FtGATA* gene family contains more acidic amino acids, suggesting that the *FtGATA* family plays a momentous role in acid-base regulation of Tartary buckwheat. It was predicted that 26 FtGATAs were localized in the nucleus, whereas the FtGATA09 and FtGATA13 proteins are in the chloroplast, considering that their function may be involved in physiological and biochemical regulation of chlorophyll synthesis.

The 28 *FtGATA* gene structures were classified into four subfamilies (Figure 1A): subfamily I (13), II (7), III (4), and IV (4), according to the *GATA* family grouping of the model plant, *Arabidopsis thaliana*. Subfamily I had the most members (46.43%), which is consistent with previous findings [40], suggesting that these *FtGATA* genes may have strong differentiation ability during long-term evolution. The 28 FtGATA protein domains in this study are highly conserved, and all contain one GATA domain; the conserved domain of four protein members in subfamily III is CX_2_CX_20_CX_2_C, whereas the conserved domain of subfamilies I, II, and IV is CX_2_CX_18_CX_2_C (Figure 1B); these results are similar to those reported by Shi et al. [40]. FtGATA proteins are distributed in different conserved domains among different subfamilies, and there are differences in the sites of different FtGATA proteins within the same subfamily. This may lead to the diversification of FtGATA proteins and different physiological and biochemical regulatory functions. At the same time, some residues among different subfamilies are highly conserved, such as TP, GP, and CNAC (Figure 1B). This suggests that they may be necessary for FtGATA function in different subfamilies.

The developmental evolutionary tree, conserved motifs, and gene structures of 28 FtGATA families were constructed to further explore their structural characteristics (Figure 2A–C). Twenty-eight FtGATA proteins had motifs 1 and 3 (Figure 2B), which suggested that these conserved motifs are necessary for FtGATA protein function. Different motifs are distributed among different subfamilies, and the same subfamily contains similar motifs (Figure 2B). This indicates that the GATA family has differentiated and evolved in different directions owing to many uncertain factors and assumed different functions. Subfamilies III and IV are rich in introns and CDS segments, especially the *FtGATA04* of subfamily III, which contains nine introns and CDS segments, respectively; however, *FtGATA02* and *FtGATA16* have no introns (Figure 2C). Introns can increase the probability of recombination between genes by increasing the length of the gene, whereas genes without introns cannot be spliced and separated and can continuously encode amino acids [41]. Intronless genes quickly respond to stress by delaying regulatory responses, thereby promoting plant growth and development [42,43].

Cis-elements are short sequences located in the gene promoter region in front of the protein-encoding sequence ATG, which regulate the activity of target genes by being activated by trans-acting elements [44]. Common cis-acting elements include hormone responses, environmental regulation, and growth and development. This study found that light-responsive elements were the most widely distributed (371) cis-acting element of the identified 28 *FtGATA* genes, followed by responsiveness elements (77) (Figure 3). GATA transcription factors regulate light signal transduction by binding to the light-responsive elements of the *GATA* gene promoter sequence [45]. Light induces the expression of *CrGATA1* and activates the promoters of five light-responsive pathway genes in plant cells [44]. *GATA2* regulates transcriptional regulators of light and biosynthetic metabolism [22]. In this study, ABA, auxin, GA, MeJA, and SA responsiveness elements were distributed in each promoter sequence (Figure 3), indicating that 28 *FtGATA* genes have physiological metabolic responses in response to hormone stress. In addition, the promoters of *FtGATA03* (subfamily II) and *FtGATA10* (subfamily I) contain a flavonoid biosynthetic gene regulation element (Figure 3), indicating that these two genes may be directly- or indirectly associated with the metabolic function of the flavonoid synthesis pathway in Tartary buckwheat.

### 3.2. Evolution of FtGATA Genes

Gene amplification is a method that can increase the generation frequency of new genes and the evolution of species and enhance the ability to adapt to the environment. [46]. A tandemly repeated gene pair (*FtGATA04* and *FtGATA05*) was identified amongst the 28 *FtGATA* genes from subfamily III (Table S6). This indicated that tandemly repeated genes may be replicated in plants during evolutionary differentiation and retain their copy numbers at a high frequency. Analysis of the fragment repeatability of 28 *FtGATA* genes on chromosomes identified eight pairs of repetitive events, among which the majority (4) of the *FtGATA* genes are part of subfamily I, and the remaining four pairs were evenly distributed in subfamily II and IV (Figure 4B, Table S7). This shows that these genes are linked in their respective subfamilies, and most of the gene structure and function information is retained after the entire genome replicates and evolves; however, there will be deviations during retention. The genomes of different subfamilies have different probabilities of tandem duplication, indicating that genes of different subfamilies differ in structure, function, environmental adaptation, mutation, and recombination. In addition, 28 *FtGATA* genes were used to analyze interspecific collinearity with six monocotyledonous and dicotyledonous plants (Figure 5). Tartary buckwheat is a dicotyledonous plant, and the *FtGATA* gene family has more collinear gene pairs with dicotyledonous plants, especially *Glycine max* (46 pairs), compared with monocotyledonous plants, especially *Oryza sativa* (one pair). In addition, the *FtGATA07* gene is co-linear with dicotyledonous and monocotyledonous plants (Figure 5 and Table S8). This indicates that *FtGATA07* has homologous genes before the differentiation of monocotyledonous and dicotyledonous plants, and the *FtGATA* gene clustered more closely with the *GATA* gene of *Glycine max* and *Vitis vinifera* dicotyledons (Figure 6), indicating that they are closely related.

### 3.3. Spatiotemporal Expression Patterns of FtGATA Genes

The overexpression and mutation of GATA transcription factors GNC and GNL in *Arabidopsis* can alter the regulation of flowering time and cold tolerance through inhibition of the GA-signaling pathway downstream of DELLA and PIF [47,48]. *GATA* genes can regulate the growth and development of fruit flowering, stress, and hormone responses and can play a significant role in plant growth and development [49]. This study explored the spatiotemporal expression level and patterns of eight *FtGATA* genes in different subfamilies, including spatial and temporal expression in grain filling, fruit development, adversity stress, and hormone response. Expression levels of most genes were high in the stems of Tartary buckwheat seedlings (Figure 7A), with *FtGATA06* and *FtGATA13* (from subfamily IV) expressed in the stems and husks of grain-filling tissues (Figure 7A), fruit in early grain filling, and husk in middle grain filling during fruit development (Figure 7C). There were high expression levels of *FtGATA23* and *FtGATA24* in flowers during the grain-filling stage (Figure 7A) and in fruits at the late and early grain-filling stages. This indicated that the development of the plant flowering period has a positive effect on fruit development. Most genes had high expression levels in leaves following 24 h NaCl treatment (Figure 8A), and most genes were significantly upregulated in leaves following 12 h PEG treatment (Figure 8A). Changes in *FtGATA06* and *FtGATA13* were similar, except for the heat treatment (Figure 8A). The expression levels of a great majority of genes in the stem was high following 3 h of hormone treatment (Figure 9A), except for *FtGATA10* and *FtGATA13*, which was similar to the results at the grain-filling stage (Figure 7A). *FtGATA24* expression was high in the leaves of plants treated with MeJA, GA, and SA. *AtGATA3* from *A. thaliana* has high homology to *FtGATA24* and regulates shoot apical meristem organization, flowering and development, hormone regulation, and other functions [50]. FtGATA24 lacked motif 9 from the conserved motifs of other protein members of subfamily I (Figure 2B), and both had collinear genes with dicotyledonous plants (Figure 5). Correlation analysis showed that most genes were significantly or not significantly positively correlated (Figure 7B,D, Figure 8B, and Figure 9D).

## 4. Materials and Methods

### 4.1. Materials, Experiment Design, and Treatments

The Tartary buckwheat variety ‘Chuanqiao-2’ used in this experiment was provided by the Alpine Crop Research Station (102.20 E, 27.96 N) of Xichang Institute of Agricultural Sciences, Sichuan Province, China. Uniform-sized seeds of ‘Chuanqiao-2’ with no pests and diseases were selected, sterilized and rinsed, and then sown in a washed and sterilized Petri dish (90 mm diameter) covered with two layers of sterile, washed qualitative filter paper (9 cm diameter). This was placed in a constant temperature incubator (16 h/25 ± 1 °C during the day, 8 h/20 ± 1 °C at night, with a relative humidity of 75%) for 7 days for germination. Tartary buckwheat seedlings with uniform growth 7 days after sowing (DAS) were transplanted into pots (diameter, height = 25.5 cm, 17.5 cm) containing mixed nutrient soil (soil: substrate = 1:1) that was sterilized at a high temperature. Each pot contained three seedlings, which were placed in a culture room under specific conditions for optimal growth (16 h/25 ± 1 °C during the day, 8 h/20 ± 1 °C at night, relative humidity 75%). Tartary buckwheat seedlings at the three-leaf and one-heart stage were treated with six kinds of abiotic stresses (cold: 4 °C, dark: complete shading, flooding: whole plant, heat: 40 ℃, NaCl: 150 mmol·L^−1^, PEG6000: 25% *w/v*), and four kinds of hormone treatments (ABA: 100 μmol·L^−1^, GA: 100 μmol·L^−1^, MeJA: 100 μmol·L^−1^, SA: 100 μmol·L^−1^). Each treatment included five replicates, and the roots, stems, and leaves were collected for 0, 3, 12, and 24 h, respectively. The tissues were rapidly placed in liquid nitrogen and stored at −80 °C [2,44]. In addition, the root, stem, young leaf, mature leaf, flower, fruit, and husk were taken from growing Tartary buckwheat to the grain-filling period; the fruits and husks were taken during the early, middle, and late grain-filling periods, and the samples were stored at −80 °C [35].

### 4.2. Total RNA Extraction, Reverse Transcription PCR (RT-PCR), and Quantitative Real-Time PCR (qRT-PCR)

A 0.1 g sample of Tartary buckwheat leaves was fully ground in liquid nitrogen, and total RNA was extracted using the E.Z.N.A. Plant RNA kit (Omega Bio-tek, Inc., Norcross, GA, USA). The integrity of extracted RNA was checked using 1% agarose gel electrophoresis, and RNA purity and concentration was measured using a spectrophotometer (Beijing Kaiao Technology Development Co., Ltd., Beijing, China). RNA was reverse transcribed into cDNA using the Hiscript II Q RT Supermix for qPCR kit (Vazyme Biotech Co., Ltd., Nanjing, China) in a 20 μL reaction system.

Primer Premier 5.0 software (Premier Corporation, Vancouver, BC, Canada) was used to design specific primers for qPCR, with *FtH3* as the internal control gene (Table S3). The ChamQ Universal SYBR qPCR master mix kit (Vazyme Biotech Co., Ltd., Nanjing, China) was used with 1.0 μL template cDNA, 10.0 μL 2 × SYBR mix, and 0.4 μL of each primer, and the reaction volume was made up to 20 μL with ddH_2_O. qPCR was performed using a CFX96 real-time system (Bio-Rad, Hercules, California, USA). The relative expression levels of target genes compared to the internal control gene were calculated using the 2^−ΔΔCt^ formula [51]. Three biological replicates and three technical replicates were set up in this experiment.

### 4.3. Genome-Wide Identification of FtGATA Genes in Fagopyrum tataricum

This study downloaded the genome sequence of Tartary buckwheat from the MAKBASE website (accessed on 28 July 2021 at http://www.mbkbase.org/Pinku1/), as well as the GATA amino acid sequences of *Arabidopsis* (accessed on 29 July 2021 at https://www.Arabidopsis.org/) and rice (accessed on 30 July 2021 at http://Rice.plantbiology.msu.edu/). All possible FtGATA proteins were identified from the *Arabidopsis* amino acid sequence using BLASTp (score value ≥ 100, e-value ≤ 1e−10) [52]. Second, the hidden Markov model (HMM) file corresponding to the GATA domain (PF00032) was obtained from the PFAM database using HMMER 3.3.2, with a cutoff value of 0.01 (accessed on 2 August 2021 at https://www.ebi.ac.uk/Tools/hmmer/search/phmmer) [53]. Furthermore, batch CD-Search (accessed on 7 August 2022 at https://www.ncbi.nlm.nih.gov/Structure/cdd/cdd.shtml) and SMART (http://smart.emblheidelberg.de/) were used to determine the domains of all FtGATA proteins [54,55]. Finally, the identified FtGATA proteins were analyzed for their protein length, molecular weight (MW), isoelectric point (PI), and instability index (II) (https://www.expasy.org/). FtGATA protein subcellular localization was predicted with WoLF PSORT (accessed on 6 August 2021 at https://psort.hgc.jp/). The FtGATA protein transmembrane helix (TMH) was predicted with DeepTMHMM (accessed on 7 August 2022 at https://dtu.biolib.com/DeepTMHMM).

### 4.4. GATA Gene Structure, Conserved Motifs, Cis-Acting Elements, and Protein Interactions

Multiple sequence alignments of GATA protein domain sequences of different subfamilies of Tartary buckwheat and *Arabidopsis thaliana* were performed with MEGA 11 software using default ClustalW parameters [56]. The coding sequence (CDS) of *FtGATA* was compared with the corresponding genomic DNA sequence using TBtools v1.0987663 software to construct a GATA gene structure map [57]. The full-length sequences of the FtGATA protein family were analyzed using MEME (accessed on 7 August 2021 at https://meme-suite.org/meme/tools/meme) with ten motifs [58]. The cis-acting elements present in the promoter sequences of the *FtGATA* gene family (2000 bp upstream) were predicted using the PlantCare website (accessed on 8 August 2021 at http://bioinformatics.psb.ugent.be/webtools/plantcare/html/).

### 4.5. Chromosomal Distribution of FtGATA Genes

All *FtGATA* genes were distributed to the Tartary buckwheat chromosome using Circos based on the Tartary buckwheat genome database [59]. Analysis of *FtGATA* gene duplications was performed using the Multiple Collinearity Scan Toolkit X [60]. A dual synteny plot was used to analyze the homology of GATA genes of *Fagopyrum tataricum* and six other plants (*Glycine max*, *Vitis vinifera*, *Solanum lycopersicum*, *Arabidopsis thaliana*, *Brachypodium distachyon*, and *Oryza sativa*) [57].

### 4.6. Phylogenetic Evolution and Classification of the FtGATA Gene Family

The muscle wrapper model was used to calculate GATA amino acid sequences of *Glycine max*, *Vitis vinifera*, *Solanum lycopersicum*, *Arabidopsis thaliana*, *Brachypodium distachyon*, and *Oryza sativa* (Table S4) downloaded from the UniProt database (accessed on 9 August 2021 at https://www.uniprot.org/) and FtGATA amino acid sequences using TBtools v1.0987663 software. IQ-Tree Wrapper (bootstrap number of 1000) was used to construct the best phylogenetic tree. All identified FtGATA proteins were grouped and analyzed according to the subfamily classification of AtGATA proteins in the *Arabidopsis* model plant.

### 4.7. Statistics and Analysis

Microsoft Excel 2010 was used for data entry and statistical analysis. Analysis of variance (ANOVA) (*p* < 0.05) and multiple comparisons (Duncan) were performed using IBM SPSS Statistics software (version 26.0; International Business Machines Corporation, Armonk, NY, USA), and the results were expressed as the mean ± SD. Column charts were drawn using GraphPad Prism 7.0 (GraphPad Software, LLC, San Diego, CA, USA).

## 5. Conclusions

Twenty-eight *FtGATA* genes were identified for the first time in the Tartary buckwheat genome. The genes were divided into four subfamilies and distributed on eight chromosomes. Chromosome mapping revealed one pair of tandem duplication genes and eight pairs of segmental duplication genes. *FtGATA* had the most collinear genes with the dicotyledonous plant *Glycine max*. In addition, spatiotemporal expression patterns of eight *FtGATA* genes in different subfamilies showed that they have regulatory roles in tissue specificity, fruit development, abiotic stress, and hormone response. In summary, this study created a theoretical and scientific foundation for studying the function and stress response of different subfamilies of *FtGATA* genes.

## Data Availability

Data are contained within the article or Supplementary Materials.

## Supplementary Materials

The following supporting information can be downloaded at: https://www.mdpi.com/article/10.3390/ijms232012434/s1.
